# Development of a checklist for evaluating psychiatric reports

**DOI:** 10.1186/s12909-019-1559-1

**Published:** 2019-05-02

**Authors:** Nadine Dreimüller, Stefan Schenkel, Marlene Stoll, Cora Koch, Klaus Lieb, Jana Juenger

**Affiliations:** 1 Department of Psychiatry and Psychotherapy, University Medical Center Mainz, Untere Zahlbacher Str. 8, 55131 Mainz, Germany; 2 IMPP, Große Langgasse 8, 55116 Mainz, Germany

**Keywords:** Checklist, Psychiatric admission report, Development

## Abstract

**Background:**

Performing a psychiatric interview and documenting the recorded findings in the form of a brief psychiatric report is one of the main learning goals in the psychiatric curriculum for medical students. However, observing and assessing students‘ reports is time consuming and there are no objective assessment tools at hand. Thus, we applied an integrative approach for designing a checklist that evaluates clinical performance, as a tool for the assessment of a psychiatric report.

**Methods:**

A systematic review of the literature yielded no objective instrument for assessing the quality of written reports of psychiatric interviews. We used a 4-step mixed-methods approach to design a checklist as an assessment tool for psychiatric reports: 1. Development of a draft checklist, using literature research and focus group interviews; 2. Pilot testing and subsequent group discussion about modifications resulting from the pilot testing; 3. Creating a scoring system; 4. Testing for interrater-reliability, internal consistency and validity.

**Results:**

The final checklist consisted of 36 items with a Cronbach’s alpha of 0.833. Selectivity of items ranged between 0.080 and 0.796. After rater-training, an interrater-reliability of 0.96 (ICC) was achieved.

**Conclusions:**

Our approach, which integrated published evidence and the knowledge of domain experts, resulted in a reliable and valid checklist. It offers an objective instrument to measure the ability to document psychiatric interviews. It facilitates a transparent assessment of students’ learning goals with the goal of structural alignment of learning goals and assessment. We discuss ways it may additionally be used to measure the ability to perform a psychiatric interview and supplement other assessment formats.

**Electronic supplementary material:**

The online version of this article (10.1186/s12909-019-1559-1) contains supplementary material, which is available to authorized users.

## Background

Interviewing is of central importance in medicine and psychiatry; it is a core skill in all disciplines of medical practice, but it is *the* core skill in clinical psychiatry, as it is the only method for accurately diagnosing psychiatric illnesses [1]. A proper and early diagnosis enables a timely intervention leading to a reduction of personal suffering of patients and direct and indirect social costs [2]. To ensure the early diagnosis of psychiatric illnesses, not only psychiatrists but all kind of doctors need to be equipped with the skill to diagnose psychiatric illnesses. Currently, however, mental disorders often go undetected by general practitioners, thus delaying proper treatment [3].

To address this deficiency, the CanMed’s Role “Communicator” [4] has been emphasized in the new National Competence-based Learning Objectives Catalogue for undergraduate medical education (NKLM) [5] in Germany. The NKLM integrates specific communication competences by requiring the testing of learning goals, for example: “You are able to write different forms of patient reports adequately” as part of the work package “Skills in professional doctor-patient interaction”.

The psychiatric interview is similar to the general medical interview in that both include the patient’s chief complaint, history of the presenting complaint, past history as well as social and family history [6]. Additionally, the psychiatric interview requires a formal examination of the patient’s mental status including risk assessment. Afterward, the knowledge acquired in the interview is written down and organized into the psychiatric report. Due to the importance of these skills for diagnosis and proper treatment, they are the main learning goals in the mandatory undergraduate psychiatric bedside teaching. It is essential to practice the writing of psychiatric reports for two reasons: 1. Making the students reflect the interview and structure its contents, helping them to conclude the correct diagnosis; 2. Recording the acquired information, thus enabling communication with colleagues as well as serving as a memory aid. As it is well known that assessment promotes learning, we aimed to develop a reliable and valid assessment method for psychiatric reports to consecutively enable sophisticated feedback and improve competence.

Reliable and valid measurement of clinical skills is a challenge, because it is often subjective and there is little time to perform the evaluation. However, it is an essential part of the learning process, as it is well known that assessment drives learning. Checklists are a helpful tool in this context because they outline detailed and objective criteria for specific exercises, ensuring that all important aspects are considered by focusing raters on predefined items. Additionally, they make the assessment process transparent for students and thus help to drive learning.

As the writing of a psychiatric report is an important skill for doctors and a main learning goal for medical students, the aim of this work was to construct an assessment tool to quantify the quality of psychiatric reports, with the objective to drive learning and improve the quality of reports of medical students. For the above-mentioned reasons, we focused on developing a checklist as an assessment tool.

## Methods

Approval for the study was obtained from the local ethic committee. As the quality of an evaluation checklist is highly affected by its development process [7], we followed the methodological recommendations for developing effective evaluation checklists given in literature. To summarize, checklists should be based on professional experience [7, 8], primary literature sources or peer reviewed guidelines [7] and the consensus of experts in the field of interest [9].

We decided on an integrative approach which combined different established methods, because none of the described methods in literature [7, 9, 10] appeared transferable to our question without modification.

**Fig. 1:**
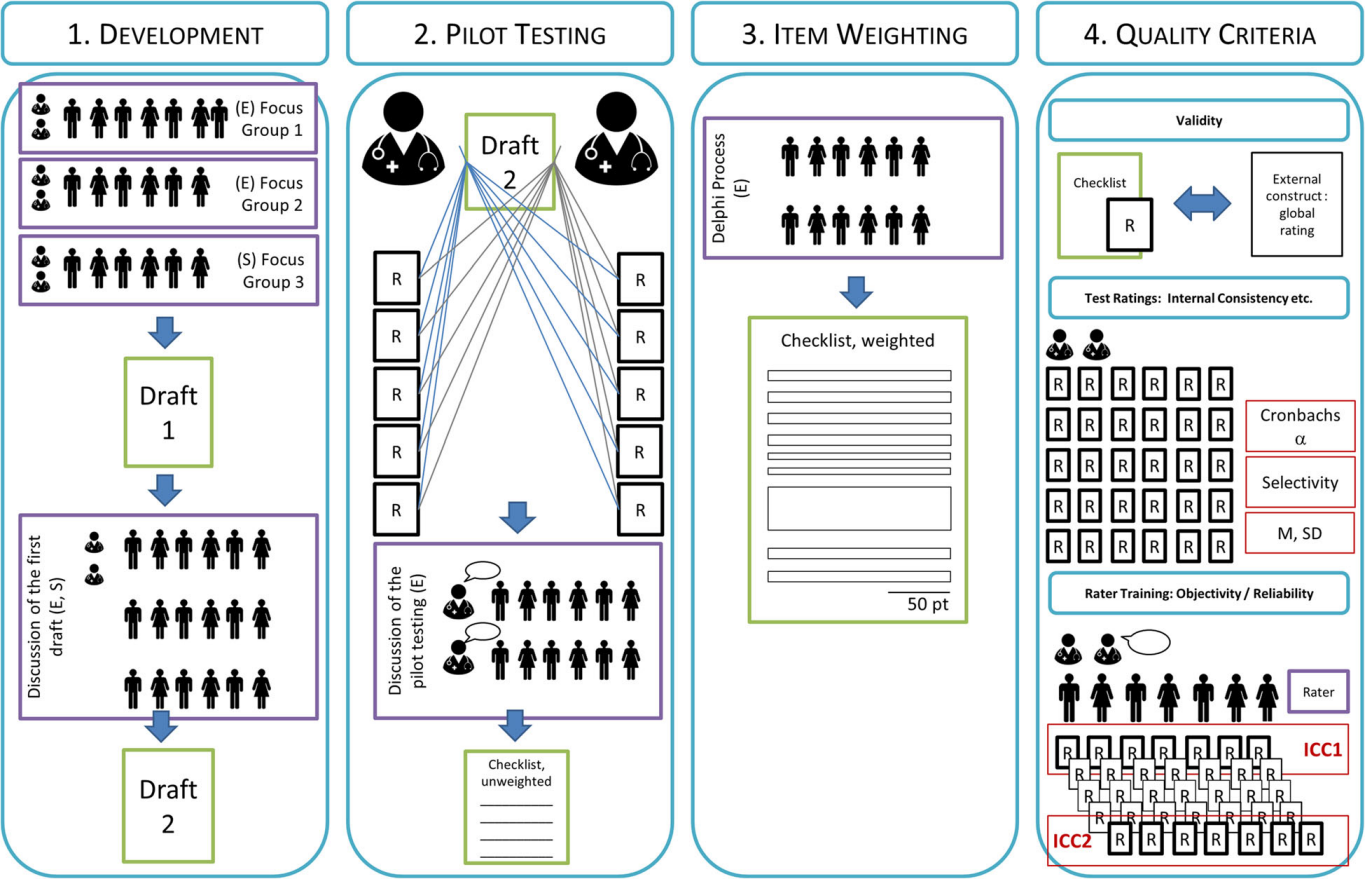
Process of checklist development

The publication that was most relevant to our research objective was the investigation of Schmutz et al. [10]. Therein, five points were suggested: 1. Development of a draft checklist; 2. Adapted Delphi review rounds; 3. Design of the final checklist and pilot testing; 4. Final Delphi round; 5. Item weighting. Since the mentioned steps could not be transferred completely to the current objective, we adapted them to our setup. Figure 1 outlines the four steps of our systematic approach for the development of performance checklists.

### Step 1: development of a draft checklist

**Fig. 2:**
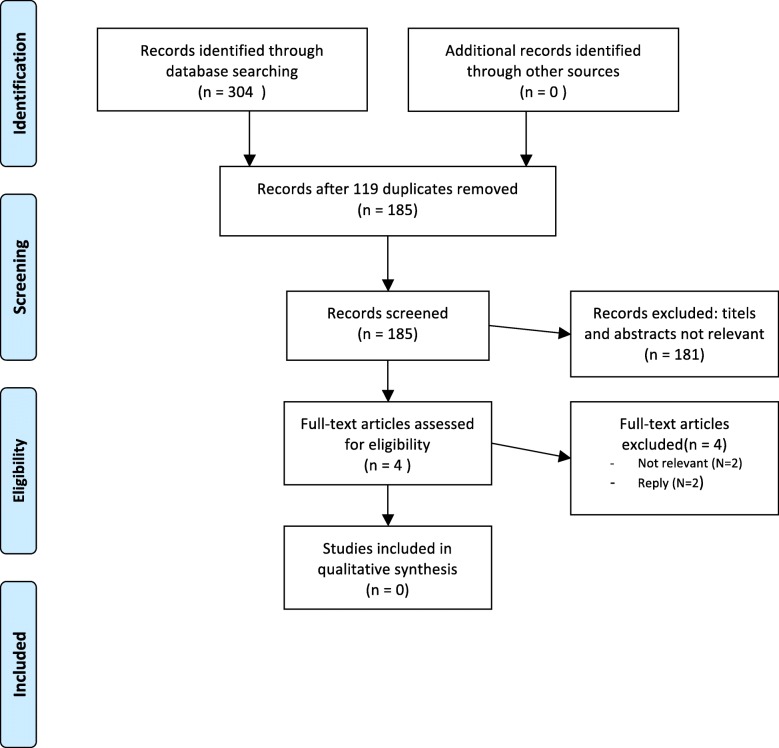
PRISMA flowchart

As proposed by Schmutz et al., we first performed a literature research via Pubmed and PsycINFO on 4th January 2015 with the search terms “checklist”, “psychiatry”, “psychopathology” and “assessment”. The search in Pubmed produced 181 results. After reviewing these abstracts, no article was found to be relevant to our objective of assessing reporting skills. PsycINFO provided 123 results, again without relevance to our objective (please see PRISMA flowchart in Fig. 2 for details [11]), although we found many publications relating to the creation of checklists, which we used to guide our process. As specific recommendations could not be found in literature we focused on clinical experience. To obtain a wide range of experience, we invited different experts to focus group interviews to discuss which items a psychiatric report definitely needs to contain.

Focus group interviews are an established technique in medical education research that has been utilized increasingly in recent years for the development of questionnaires and similar instruments [12, 13]. Thereby, information can be gathered more quickly, less resources are needed than in one-on-one interviews and the direct interactions between the participants supply additional information [14].

### Participants

Participants were chosen according to the different perspectives they could give on the objective. Experience in obtaining psychiatric reports in different settings was considered as the most important qualification. Following the recommendations for focus groups in the AMEE-Guide by Stalmeijer et al. [15], which suggest a maximum number of eight participants, and in order to cover the various sub-areas of psychiatry, we invited 19 participants for three focus groups. Participants represented the following fields: Social psychiatry, outpatient psychiatry, inpatient psychiatry, consult liaison psychiatry, gerontopsychiatry and general medicine. Due to the fact that heterogeneity in terms of hierarchy is problematic for the sense of safety necessary for collecting information and ideas from the group, we formed a separate group of medical students. Except for the students, the experts of each above mentioned field in the focus group interview had 6 to 14 years of experience in psychiatry.

### Procedure

We used a standardized focus group guide to perform focus groups. We also had a predefined set of questions (see Additional file 1) and used a systematic analysis process [17]. Following literature recommendations, we started with two consecutive focus groups and planned as many as necessary in respect of the principle of satisfaction (ceasing the procedure when there is no additional information gained) [16]. After the expert focus groups, we discussed the results with medical students in another focus group. The length of the discussion was set to 90 min, to have time for an adequate discussion while making the process viable considering the time constraints of experts active in clinical practice. The authors ND and SS developed a list of key questions in order to moderate the discussion (see Additional file 1) [17]. During the focus group the moderator offered a summary of results and sought confirmation. Immediately after the focus group, the study investigators noted themes, interpretations and ideas.

The focus groups were recorded, with consent of participants, by the App *F5 Transcription* (obtainable at autotranscription.de) and later transcribed partly digitally (likewise with *F5 Transcript*), partly by a research assistant. Additionally, interviewers’ and observers’ perceptions were documented, as well as visualizations of findings on a sheet of paper that the focus group participants worked on together. Then a report was prepared of the individual focus group. After accomplishing the focus group interviews, data was analyzed. To evaluate the interviews, we analyzed opinions of the individual participants as well as their interaction results and the visualization of findings. Later we analyzed the series of focus groups. We compared and contrasted results by categories of individual focus groups. Then findings were described. The groups served for generating ideas, and based on the results of our analysis, the study investigators developed a preliminary checklist.

In accordance with the “multiple methods” approach recommended by Schmutz et al. [9], the preliminary checklist was sent to the participants of the focus groups, inviting them to propose suggestions for changes, criticism and annotations. Those were discussed by the study investigators who then modified the checklist by decision making in consensus.

### Step 2: pilot testing

In a second step, we pilot tested the checklist. Two experienced clinicians rated 10 historic reports and suggested modifications of the checklist. Vaguely defined items as well as missing or redundant items were identified. Analogous to step 4 by Schmutz et al. [10], the original participants of the focus groups discussed the raters’ suggestions to withdraw irrelevant items or to change or add others. The results of the discussion are presented in the results section.

### Step 3 item weighting

Classic checklists use simple dichotomous items like “accomplished/not accomplished”. The problem with this approach is that dichotomous measurements are often not sufficient regarding complex skills and, in the case at issue, not every item in the checklist is equally important for obtaining a diagnosis. A checklist differentiating between essential and less important items thus provides more accurate performance assessments. Hence, in the next step, items were weighted to allow a more nuanced assessment of complex skills and to attach more importance to more relevant tasks. The participants of the focus groups were asked in a Delphi-process (a standardized method of evaluating an object with feedback loops) to apportion a certain number of points to each item [18]. The maximum number of points was set at 50. The results were discussed in our working group until a consensus on the weighting of the items was reached.

### Step 4 internal consistency, construct validity and interrater-reliability

In order to measure the convergent validity of the checklist, we applied an external construct commonly used for validation, a global expert performance rating using school marks (1–6) [19]. We then correlated the global ratings of the students’ reports with the checklist scores of the same students’ reports. For further analysis of quality criteria, ND and SS evaluated 30 student reports using the checklist. Internal consistency was examined by Cronbach’s alpha and calculation of selectivity.

To improve objectivity and reliability of the rating instrument, we developed a standardized rater training in a next step. We used interrater-reliability, measured by ICC, to assess quality of rater training [20]. The training proceeded according to a standardized pattern: Following a short introduction and exposition to the checklist, five exemplary reports were rated and the results discussed afterwards. Five interns and two research assistants were trained (2 men, 5 women) in our rater training. The average clinical experience was 2.1 years (SD 2.1, range 0–5). Prior to the training, the reports were evaluated by two doctors who were involved in the development of the checklist (ND & SS). The items were judged by a scoring system: For consistent results with the expert rating we assigned one point, for divergent outcomes no points. An accuracy of 80% was assessed as acceptable and conditions of more than 90% as highly reliable.

The findings were analyzed using SPSS 20. We calculated correlations for various variables. These were judged as significant if *p* < .05.

## Results

### Step 1 development of a draft checklist

Three focus groups were performed. The second expert focus group yielded no major new ideas, so we did not perform any further expert focus groups. In a third focus group with medical students we validated and discussed the results (see Fig. 1).

We developed a draft checklist based on the visualizations and notes of the focus group participants and the transcripts of the focus group interviews. The draft checklist consisted of 30 items. The first review round led to the rephrasing of four items and addition of three further items. The rephrasing mainly had linguistic reasons: for example “since when sick” vs. “duration of illness”, “aggressiveness” vs. “endangerment to others”. The following items were added: confirmability of statements, structure and appearance.

### Step 2 pilot testing

The clinicians who performed the pilot testing of the draft checklist noted items where adjustments were necessary to improve the application of the checklist (see Fig. 1). These points were discussed by the clinicians and some participants of the previously described focus groups. After this discussion, four items were added (endangerment to self, interactions, other diagnoses, illness insight), one item was deleted (outpatient vs. inpatient) and two items were rephrased: (old: formal thought disorder, new: thought process and old: drive disorder, new: psychomotor activity), because the description was not precise enough. The checklist then consisted of nine blocks with up to 18 items, for a total of 36 items.

### Step 3 item weighting

According to importance, a range of 1–3 points per item could be reached. We set the maximum number of points at 50. After a group discussion, we determined that half of the points should be apportioned to the mental state examination, consisting of 18/36 items, because this part is the most important when generating the correct diagnosis. Twenty-four items (66.7%) received one point, 10 items (27.8%) two points and two items (5.6%) three points.

### Step 4 internal consistency, construct validity and interrater-reliability

Cronbach’s alpha for internal consistency between items and scale was 0.83. Calculation of selectivity of items showed correlation values between *r* = 0.080 and *r* = 0.796, with the highest values in impression (*r* = 0.796) and the accurate diagnosis (*r* = 0.641). The item “presenting complaint” had the lowest value (*r* = 0.080) (see Table 1). For the original dataset for the evaluation, see Additional file 2.

**Table 1:** Checklist items with respective means, standard deviations and item-scale correlations (*N* = 30)

	Item	Max	Mean	Standard deviation	Item-scale correlation
Chief complaint		4	3.27	1.03	0.119
	Admission procedures	1	0.83	0.38	0.326
	Current complaints	3	2.43	0.86	0.080
Substance abuse		3	2.50	0.96	0.322
	Consumed substances	1	0.90	0.31	0.582
	Consumed quantity	1	0.77	0.43	0.431
	Duration of addiction	1	0.83	0.38	0.386
Biography		3	2.33	0.96	0.586
	Family status	1	0.80	0.41	0.562
	Profession	1	0.83	0.38	0.533
	Education	1	0.70	0.47	0.445
Past psychiatric history		3	2.27	0.94	0.642
	Frequency of episodes	1	0.70	0.47	0.121
	Different diagnosis (if necessary)	1	0.70	0.47	0.218
	Duration of disease (since when?)	1	0.87	0.35	0.178
Family history	Family history	1	0.77	0.43	0.134
Past medical history	Comorbidities	1	0.83	0.38	0.242
Mental State Examination		25	19.63	4.84	0.725
	Orientation	2	1.77	0.43	0.165
	Interaction	1	0.87	0.35	0.217
	Attention/concentration	1	0.87	0.35	0.296
	Sleep	1	0.73	0.45	0.037
	Appearance	1	0.50	0.51	0.243
	Memory	1	0.73	0.45	0.411
	Thought process	2	1.60	0.72	0.211
	Fears	1	0.67	0.48	0.650
	Compulsions	1	0.53	0.51	0.368
	Delusion	2	1.83	0.46	0.130
	Perceptual disturbances	2	1.73	0.64	0.414
	Self-disorders	2	1.53	0.82	0.321
	Affect	2	1.67	0.61	0.578
	Psychomotor activity	1	0.90	0.31	0.357
	Suicidality	2	1.87	0.43	0.257
	Endangerment to self	1	0.73	0.45	0.473
	Endangerment to others	1	0.63	0.49	0.348
	Illness insight	1	0.70	0.47	0.346
Diagnosis		5	3.43	1.33	0.554
	Correct diagnosis	3	2.60	0.81	0.641
	Explained why making this diagnosis	2	0.87	0.86	0.320
Impression		5	3.03	1.67	0.526
	Confirmability of statements	1	0.70	0.47	0.445
	Structure	2	1.30	0.92	0.548
	Coherence (at rater’s discretion)	2	1.03	0.72	0.796

Analysis of convergent validity in the form of a correlation between the global ratings of the independent experts using school marks (1–6) and the sum results of the checklist showed a medium to high correlation with a correlation coefficient of *r* = 0.62 *p* < .001.

The interrater-reliability had already been high in the first rating of the rater training (ICC = 0.82) and rose until the fifth rating to ICC = 0.96. The most problematic items were: “confirmability of the statements” as well as “coherence” (both in block 9: global rating). “Confirmability” meant that, with the information in anamnesis and mental state examination provided, the concluded diagnosis is comprehensible. The item “coherence” was at the discretion of the rater. Here they could give points for the impression. However, these items could be improved after explanation and discussion. Regarding the different levels of clinical experience of raters we found no differences in checklist scores.

### Final checklist

The final checklist consists of 36 items, structured in nine parts. The parts are defined as follows: 1. Chief complaint; 2. Substance abuse; 3. Biography and social history; 4. Past psychiatric history; 5. Family history; 6 Past medical history; 7. Mental status examination; 8. Diagnosis; 9. Impression (see Table 1).

Every part contains between one (family history and somatic history) and a maximum of 18 items (Mental State Examination) (see Table 1). Items are weighted according to their importance. For items consisting of only one point, the correct and complete description of the item leads to one point. In items consisting of more than one point, the number of points depends on the quality of the answer, i.e. the description of current complaints can be rated on a scale from zero to three points depending on the quality of the description. In the event that patients refuse to give information regarding specific questions or there is no abnormality found, the student has to note this in order to gain points for this item. In the 30 test ratings the SD for the items with one point (M = 0.714) varied between 0.30 and 0.57. For items with two points (M = 1.45), the SD varied between 0.43–0.91. For items with three points (M = 2.5), the SD varied between 0.83–0.86. See the final checklist in Additional file 3.

## Discussion

Previous to this work, no objective measurement tool existed to assess psychiatric reports, although many checklists exist to measure the quality of psychiatric interviews via OSCE or Mini-Cex. Through a highly complex multimethod approach, we therefore developed a checklist for evaluating psychiatric reports. Following, we discuss the strengths and weaknesses of the checklist; give an overview of the challenges the developmental process offered; discuss the possible applications of the checklist; and look at the next steps that might be of interest.

The developed checklist is easy to use and viable in routine. Time demands for implementation are manageable and duration of ratings is estimated at about 5 min after some practice. This makes it a promising alternative to a global rating of the reports, which is currently common practice and is difficult for young colleagues. The interrater-reliability of the checklist is high (0.96) and no differences were found in different levels of clinical experience of raters, especially for raters with little clinical experience, it improves reliability.

The scale itself has a high internal consistency (Cronbach’s alpha = 0.83). In 70% of the items the selectivity exceeds 0.3 and only a few items showed lower selectivity. One possible explanation of the low selectivity of some items may be the ceiling effect. For example, for “current complaints”, 70% of the students reached the full score. The highest selectivity values were found in impression (0.796) and correct diagnosis (0.641). This last correlation between the item “correct diagnosis” and the whole scale shows the well-established fact that you need all the available information a patient is giving you to make a valid diagnosis. This correlation is another important indication for high construct validity.

Contrarily, we did not expect the high selectivity of the block “impression”. Beforehand, most discussions in the focus group were about the use of “impression” as block and “coherence” (at the rater’s discretion). Some of the focus group members thought this was unnecessary in a scale that assesses different aspects that should result in a general impression. Although an important role is attributed to impressions in literature [21], one should not underestimate the problems in rater training and reliability. In the case of this checklist, however, no problems in rater trainings and reliability occurred. The fact that “impression” shows such a high correlation with the scale should not lead to the conclusion that a more detailed assessment of single items is not necessary. Contrarily, it is possible that the high correlation is due to the given anchor points for the raters. With these anchors given the global assessment of competence is improved.

There are some limitations concerning the transferability of the checklist to other areas. As medical guidelines vary on a regional or national basis the checklist cannot be adopted in other countries without further adjustments [22]. For example, whereas self-disorders are a separate item of the mental status examination in Germany, they are categorized under “delusions” in Anglo-American regions. Furthermore, there may be parts that are found to be missing by some or certain parts that are found to be redundant by others. So, there may be differences in selection and weighting of specific contents that stand in the way of a one-to-one applicability to different cultures. Another problem mentioned in the current phase of using the checklist in daily routine is that there is no opportunity for free text to address special features of the report. These points should be considered by teachers who want to implement the checklist in their curriculum.

Areas for application of the checklist lie, first of all, in education. With the checklist, one can assess the students’ performance in writing a psychiatric report, give them specific feedback, and at the same time show them which points are important for a successful interview. According to the concept of “assessment drives learning” [23], the checklist may lead to a better performance of students in writing reports or even in conducting interviews.

This raises the question if the checklist may not only be used to measure the competence of writing the report, but also to measure the competence of performing the interviews. The report shows whether a student has collected all relevant information and knows how to interpret it, which is the essential part of performing the interview. Furthermore, in practice, the reason why the report is of high importance for doctors, is that it is a tool to communicate with other doctors who take a look in the patient chart. It can also function as a memory aid for the doctor him or herself, helping him remember the important details of the interview. Without a good report, an excellent interview is of little use, as the gained information is lost. On the other hand, there are other skills besides the gathering of information in an interview that could be evaluated, as for example communication skills or empathy. A very good report can result from a cold, unpleasant interview. Only few studies have assessed whether a checklist rating a psychiatric report can be used to assess the performance during an interview, but these studies showed a close correlation between the quality of interviews and the write-up [24–26]. This leads to the conclusion that it may be possible to assess psychiatric interviewing with this method as well. Further investigations are necessary to investigate this theory. If the assumed correlation can be confirmed, it will be a helpful tool and broaden the possibilities of assessment.

Although we are at an early point of development and further research is needed, we see the chance of transferability of our checklist to many contexts of assessment of psychiatric interviews, as we used a multiple methods approach and had a large diversity of participating experts. Besides teaching, the checklist may be used by young doctors or novices themselves and give them security in everyday-tasks.

On a meta-level, the checklist can serve as an instrument to question one’s own teaching style and, as appropriate, to adjust it. By using a quantitative measuring instrument, teachers are better able to evaluate the achievement of learning goals, as the detailed evaluation using the checklist can help understand which parts of the learning goals are not fully accomplished. In the next step, the teaching contents or the methods can be better coordinated with the learning goals. Through the development of a quantitative measuring instrument, a constructive alignment between learning goal, content and examination is achieved.

The development of this checklist was just a first step. The next step is to examine if the checklist improves students’ ability to write reports or even their ability to conduct an interview. Furthermore, its transmission in other medical or cultural areas should be examined. Especially in countries with small financial resources for elaborate assessment formats as OSCEs for example this cost effective tool can be helpful and may supplement the existing assessment tools.

Finally, we think that its use as an indicator for performance is a promising idea that should be evaluated and considered in further discussions about exams in psychiatry.

## Conclusions

The developed checklist promises to be a reliable and valid method that assesses the quality of psychiatric reports and is easy to implement in teaching routine.

In contrast to a global rating, it facilitates assessing the competence to document psychiatric history, giving specific feedback to students and identifying weaknesses in the curriculum. Assessing students’ performance in written findings may even be a new approach to test for clinical competence, supporting a workplace based assessment. We therefore think the checklist can improve teaching and assessing the competence to take and document psychiatric history.

## Additional files

Additional file 1: Key questions of focus group interviews. (PDF 7 kb)

Additional file 2: Dataset checklist. (XLSX 14 kb)

Additional file 3: Original Checklist. This file shows the developed checklist. (PDF 136 kb)

## Abbreviations

AMEE: The Association for Medical Education in Europe
ICC: Intraclass Correlations
M: Mean
NKLM: Nationaler Kompetenzbasierter Lernzielkatalog Medizin (German National Competence-Based Learning Objectives for Undergraduate Medical Education)
SD: Standard deviation
