# Supplementary material for: Development of a checklist for evaluating psychiatric reports
Source: BMC Med Educ. 2019 May 2;19:121. doi: 10.1186/s12909-019-1559-1 (PMC6498494; doi:10.1186/s12909-019-1559-1)
Supplement: Supplementary file 1 — Key questions of focus group interviews. (PDF 7 kb) [file 12909_2019_1559_MOESM1_ESM.pdf]

Key questions of focus group interviews

1. What content is essential to the report?
2. Which aspects are of particular importance in your specialty?
3. How should findings be structured?
4. Which headings should be used?
5. How would you define the items?
6. What is most relevant and what negligible?
7. How should subsections be structured?
8. Should there be a global rating?
9. Which points should be added here?
10. Is there anything we forgot?
