# Supplementary material for: Development of a checklist for evaluating psychiatric reports
Source: BMC Med Educ. 2019 May 2;19:121. doi: 10.1186/s12909-019-1559-1 (PMC6498494; doi:10.1186/s12909-019-1559-1)
Supplement: Supplementary file 3 — Original Checklist. This file shows the developed checklist. (PDF 136 kb) [file 12909_2019_1559_MOESM3_ESM.pdf]

	Topic	Points	
		Available points	Gained points
**1: Chief complaint**	Admission procedures	*(1 point)*	
	Current complaints	*(3 points)*	
		**In total** *(max. 4 points)*	
**2: Substance abuse**	Consumed substances	*(1 point)*	
	Consumed quantity	*(1 point)*	
	Duration of addiction	*(1 point)*	
		**In total** *(max. 3 points)*	
**3: Biography**	Family status	*(1 point)*	
	Profession	*(1 point)*	
	Education	*(1 point)*	
		**In total** *(max. 3 points)*	
**4: Past psychiatric history**	Frequency of episodes	*(1 point)*	
	Different diagnosis (if necessary)	*(1 point)*	
	Duration of disease (since when?)	*(1 point)*	
		**In total** *(max. 3 points)*	
**5: Family history**		**In total** *(max. 1 point)*	
**6: Past medical history**	Comorbidities	*(1 point)*	
		**In total** *(max. 1 point)*	
**7: Mental State Examination**	Orientation	*(2 points)*	
	Interaction	*(1 point)*	
	Attention/concentration	*(1 point)*	
	Sleep	*(1 point)*	
	Appearance	*(1 point)*	
	Memory	*(1 point)*	
	Thought process	*(2 points)*	
	Fears	*(1 point)*	
	Compulsions	*(1 point)*	
	Delusion	*(2 points)*	
	Perceptual disturbances	*(2 points)*	
	Self-disorders	*(2 points)*	
	Affect	*(2 points)*	
	Psychomotor activity	*(1 point)*	
	Suicidality	*(2 points)*	
	Endangerment to self	*(1 point)*	
	Endangerment to others	*(1 point)*	
	Illness insight	*(1 point)*	
		**In total** *(max. 25 points)*	
**8: Diagnosis**	Correct diagnosis	*(3 points)*	
	Explained why making this diagnosis	*(2 points)*	
		**In total** *(max. 5 points)*	

**9: Impression**	Confirmability of statements	*(1 point)*	
	Structure	*(2 points)*	
	Coherence	*(2 points)*	
		**In total** *(max. 5 points)*	
		**Total sum MAX 50 Points**	

Additional File 4 Checklist to evaluate the report
